# SFRP2 and Slug Contribute to Cellular Resistance to Apoptosis in Hypertrophic Scars

**DOI:** 10.1371/journal.pone.0050229

**Published:** 2012-12-04

**Authors:** Liang Chen, Zhenxiang Wang, Shirong Li, Guangjian Zhao, Maosheng Tian, Zhicheng Sun

**Affiliations:** 1 Department of Plastic Surgery, Southwest Hospital, Third Military Medical University, Chongqing, P. R. China; 2 Department of Plastic Surgery, The Affiliated Hospital (Pingjin Hospital), Logistical College of Chinese People’s Armed Police Force, Tianjin, P. R. China; UT-Southwestern Med Ctr, United States of America

## Abstract

Hypertrophic scars (HS) are skin disorders which occur after wounding and thermal injury. Our previous studies have suggested that secreted frizzled-related protein 2 (SFRP2) is involved in HS formation and that the suppression of SFRP2 promotes apoptosis of hypertrophic scar fibroblasts (HSFBs). However, the mechanisms have not been clarified. Previous studies revealed that Slug expression inhibits cell apoptosis, in vitro and in vivo, and SFRP2 regulates the expression of Slug in cervical cancer cells. In the present study, we quantified differential expression levels of expression of SFRP2 and Slug in HS and normal skin tissues by immunohistochemistry, both of which have important anti-apoptosis roles. Furthermore, a short hairpin RNA approach was adopted to investigate the potential function of SFRP2 and Slug in HSFB apoptosis. Cell apoptosis was detected using fluorescence-activated cell sorting and Caspase-3 activity was assayed by spectrophotometry. This study demonstrates that SFRP2 expression, as well as Slug, is dramatically up-regulated in HS relative to normal skin tissues, and the Slug expression is positively correlated with SFRP2. Slug expression was down-regulated in SFRP2-deficient cells, and the down-regulation of Slug expression increased sensitivity to apoptosis which was induced through a caspase-3-dependent pathway. The infected cells with reduced levels of Slug were tested for the expression of apoptosis-related genes (Bcl-2, Bax and PUMA) which were previously identified as Slug targets. Bcl-2 expression was down-regulated in Slug-deficient cells. In conclusion, SFRP2 appears to interact with Slug to affect the apoptosis of hypertrophic scar fibroblasts.

## Introduction

Hypertrophic scars (HS) are fibroproliferative disorders in the wound healing process and result in severe functional and esthetic defects. HS are characterized by excessive deposition of fibroblast-derived extracellular matrix (ECM) proteins, especially collagen, which result from abnormal fibroblast apoptosis and proliferation [1]. Histologically, in hypertrophic scars, orientated collagen bundles are flatter, with the fibers arranged in a wavy pattern but predominantly bundle orientation parallel to the epithelial surface [2]. The ideal consequence of human wounding is functional and scarless healing [3]. However, this rarely occurs, HS are among the most common and frustrating problems after injury. Poor understanding of the underlying mechanism has impeded research progress in this disease [4].

HS are characterized histologically by an abundance of collagenous scar tissues and an accumulation of fibroblasts which may result from abnormal fibroblast proliferation and apoptosis [1]; [5]. Despite a lack of insight into the mechanism of HS, decreased fibroblast apoptosis has long been hypothesized to be the key pathological event in the formation of HS [6]; [7]. Increasingly, the roles of several apoptosis-related genes including Bcl-2 [8], Bax [9] and p53 [10], have been recognized.

Secreted frizzled-related protein 2 (SFRP2) is a secreted glycoprotein molecule containing an N-terminal domain which is typically 30–50% identical to the putative Wnt-binding site of the frizzled receptor and a C-terminal heparin-binding domain with weak homology to netrins. SFRP2 structurally resembles cell surface frizzled receptors but lacks the transmembrane domain. It has been implicated in diverse cellular processes such as cell apoptosis regulation and differentiation [11]. In previous studies, we demonstrated that SFRP2 was significantly increased in HS tissue [12], and it acts in HS formation through suppressing of fibroblasts apoptosis [13]. However, the underlying mechanism is not clear.

Slug (Snail2), a member of the Snail family of zinc-finger transcription factors, exhibits an anti-apoptotic effect through regulation of the Bcl-2 and Bax expression and the transactivation of Puma (p53-up-regulated modulator of apoptosis) [14]; [15]. An important fact is that, in the fibroblast-like synoviocytes of rheumatoid arthritis, Slug suppression induces apoptosis through Puma transactivation [16]. Another study [17] demonstrated that altered gene expression consistent with a role for Slug in keratinocyte development and apoptosis was evident in epidermis from Slug-null mice. Although the roles of Slug in apoptosis are being clarified in many tumours, the epidermis, and fibroblast-like synoviocytes, little progress has been made in elucidating the mechanism underlying Slug regulation in HS.

Recently, Chung et al. [18] reported a regulatory role for SFRP2 in Slug expression in cervical cancer cells. However, the relationship between SFRP2 and Slug in HS formation was not addressed. In the present study, our approach was to investigate the Slug expressions in skin fibroblasts, scars and hypertrophic scar tissues using immunohistochemistry and to evaluate whether these are related to HS formation. Moreover, we hypothesize that short hairpin RNA (shRNA) targeting SFRP2 can influence apoptosis through altered Slug expression. To investigate the apoptosis signal in HS formation, the Bcl-2, Bax and PUMA expressions were evaluated in the context of Slug down-regulation by RNA interference. We thus investigated the roles of SFRP2 and Slug in the regulation of fibroblast apoptosis in HS formation and also indicated a potential mechanism by which targeted SFRP2 or Slug therapy may be effective as anti-scarring therapeutic agents.

## Methods

### Patient Specimens

Normal human skin and hypertrophic scars were obtained from the Department of Plastic Surgery at Southwest Hospital (Third Military Medical University, Chongqing). Thirty-eight patients (21 men and 17 women, aged 15–38 years) provided tissue for immunohistochemistry. The lesions were diagnosed as hypertrophic scars on the basis of clinical appearance and histopathological examination results at 6–12 months after the burn injury. Fifteen hypertrophic scars were located on the upper arm, 9 on the face, 9 on the upper front of the shoulder, and 5 on the anterior portion of the chest. Normal skin, provided by 22 patients (10 men and 12 women, aged 21–40 years), was obtained from the thigh region during surgery. The 9 fresh specimens for RT-PCR and western blotting were obtained from 6 patients (Table 1). Informed consents were provied by all the subjects or their relatives. The studies were approved by the local ethics committee.

**Table 1:** Patient characteristics.

Case	Fresh Tissue	Primary Cell	Sex	Age (yr)	Anatomic location	Ethnicity	Normal skin	Hypertrophic scar
1	Yes	No	F	22	Upper arm	China	Yes	Yes
2	Yes	No	M	32	Anterior portion of the chest	China	Yes	Yes
3	Yes	No	M	33	Upper front of the shoulder	China	Yes	Yes
4	Yes	No	F	24	Face	China	Yes	No
5	Yes	No	F	25	Anterior portion of the chest	China	Yes	No
6	Yes	No	M	33	Upper arm	China	No	Yes
7	No	Yes	M	20	Upper arm	China	Yes	Yes
8	No	Yes	F	38	Upper front of the shoulder	China	Yes	Yes
9	No	Yes	F	30	Upper front of the shoulder	China	Yes	No
10	No	Yes	F	32	Anterior portion of the chest	China	No	Yes

* Tissues for semi-quantitative RT-PCR and Western blot.

### Immunohistochemistry

The immunohistochemical study was performed on alcohol-formalin-fixed paraffin-embedded sections, as previously described [19]. The surgical specimens were fixed in 4% paraformaldehyde and embedded in paraffin. Five-micrometer-thick sections were de-waxed and rehydrated, and then boiled in antigen-retrieval solution (0.01 mol/L sodium citrate buffer, pH 6.0) at 120°C for 10 min. After treatment with 3% hydrogen peroxide and incubation with 3% bovine serum albumin (BSA) to block nonspecific binding the sections were incubated with rabbit anti-SFRP2 antibodies (Abcam, Cambridge, UK) at a dilution of 1∶100, or with rabbit anti-Slug antibodies (Abcam) at a 1∶1000 dilution, or with rabbit anti Bcl-2 (Cell Signaling Technology, Boston, MA) at 1∶500 dilution for 20 h at 4°C. As negative controls, the primary antibodies were replaced with 3% BSA. After washing, the sections were treated with biotinylated goat anti-rabbit immunoglobulin G (IgG, Jackson ImmunoResearch, West Grove, PA, U.S.A) at a 1∶200 dilution and an avidin-biotin peroxidase complex (Vector Laboratories, Burlingame, CA) for 2 h at room temperature. Peroxidase activity was subsequently detected with diaminobenzidine and 0.15% hydrogen peroxidase. Counterstaining was performed with hematoxylin.

The immunohistochemical evaluation was independently performed by 2 investigators. The sections were examined under light microscopy. The number of positive cells was counted under ×400 magnification in 5 fields per section and 3 sections from each sample. Positive percentages for SFRP2 and Slug were determined by semi-quantitative optical analysis, using the product of the percentage of positive cells.

### Primary Cell Culture

HSFBs were established as primary cell lines either from normal skin or from HS tissue obtained from patients (Table 1) whom were recovering from severe burn at the department of plastic surgery, southwest hospital, third military medical university, chongqing. The diagnoses of hypertrophic scars were according to information provided by histological assessment and clinical appearance. The primary culture was prepared by an enzymatic digestion procedure, as described previously [13]. In brief, normal skin or HS tissue was minced to a size of approximately 1×1×0.5 cm. After washing with phosphate-buffered saline (PBS) containing 1% penicillin, amphotericin B, and streptomycin sulphate, the tissues were treated overnight (37°C for 6 h) with a mixture solution of collagenase type I (0.5 mg/mL) and trypsin (0.2 mg/mL). The primary fibroblasts were then cultured in Dulbecco’s modification of Eagle,s minimum essential medium (Gibco Invitrogen Corporation, Carlsbad, CA) at 37°C in a humid incubator with 5% CO_2_.

### RNA Extraction and Semi-quantitative RT-PCR

Total RNA was isolated from 9 fresh specimens consisting of 5 normal skin and 4 HS tissues with an RNAprep pure tissue kit (Tiangen, Beijing, China), according to the manufacturer’s instructions. RNA was extracted from the cultured primary fibroblasts using TRIzol (Invitrogen, Carlsbad, CA, USA) and treated with DNase I. The cDNA was synthesised from 1 µg of total RNA in a 20 µL reaction mixture volume using a reverse transcription system (Takara, Bio Inc, Shiga, Japan) with random hexamers. PCR was performed using primers specific for each target gene, and β-actin served as an internal control of the resection. The conditions were chosen such that all of the amplification products obtained from the RNAs of interest were formed during the exponential phase. The primer pairs were synthesis to target specific cDNA segments of Slug, Bcl-2, and β-actin, as described [19], and primers for Bax and PUMA were designed using the Primer3 online software. The primers sequences were 5′-AAACTACAGCGAACTGGACA-3′′ and 5′-GTCTGGAAAAC GCCTTGC-3′ for *Slug*, 5′-TATCCAATCCTGTGCTGCTAT-3′ and 5′-CTCTTG CGGAGTATTTGTGC-3′ for *Bcl-2*, 5′-TCTGACGGCAACTTCAACTG-3′ and 5′-AGGAAAACGCATTATAGACCAC-3′ for *Bax*, 5′-GCGGACGACCTCAAC GCACAGT-3′ and 5′-CGGGCAGAGCACAGGATTCACA-3′ for PUMA, 5′-GCCATGTACGTAGCCATCCA and 5′-GAACCGCTCAATGCCGATAG-3′ for *β-actin*. A 10 µL PCR product was electrophoresed on a 1% agarose gel containing ethidium bromide, and gels were visualised and photographed under UV light.

### Western Blotting

Extracts equivalent to 20 µg of total protein from the cell samples and 9 fresh tissues were subjected to 12% SDS-PAGE and then transferred onto polyvinylidene diﬂuoride membranes (Invitrogen Corporation). After blocking with 5% defatted milk in TBS containing 0.1% Tween-20, the membranes were incubated with primary antibodies against SFRP2 (1∶200; Abcam), Slug (1∶1000; Abcam), Bcl-2 (1∶2000; Cell Signaling Technology, Boston, MA), Bax (1∶1000; Cell Signaling Technology), PUMA (1∶1000; Cell Signaling Technology), and β-actin (1∶500; Santa Cruz Biotechnology), respectively. The membrane was further incubated with horseradish peroxidase-conjugated IgG (1∶2000; Santa Cruz Biotechnology) for 2 h at room temperature. The bound antibodies were detected with ECL Western Blotting Analysis System (Pierce, Rockford, IL). The relative densities of SFRP2, Slug, Bcl-2, Bax and PUMA were determined by normalisation against β-actin.

### Silencing of SFRP2 and Slug by shRNA

Three pairs of shRNAs targeting different regions of the human SFRP2 transcript (GenBank: NM_003013) and 1 control shRNA were designed and synthesised (Augct, China) and the experiments were performed in as our previous study [13].

The siRNA targeting Slug (Slug siRNA, sc-38393) and mock siRNA (mock, sc-37007) were obtained from Santa Cruz Biotechnology. Mock siRNA is a non-targeting 0- to 25-nt siRNA designed as a negative control. In a 6-well tissue culture plate, 2×10^5^ fibroblasts were seeded per well in 2 mL of antibiotic-free normal growth medium supplemented with FBS, and then incubated at 37°C in a CO_2_ incubator for 20 h until the cells were 60–80% confluent. The RNA interference was performed according to the manufacturer’s instructions. The cell were assayed by RT-PCR, western blotting, and fluorescence-activated cell sorting (FACS) on the second day after the transfection.

### Cell Apoptosis Analysis by Flow Cytometry

HSFBs transfected with target shRNA or control shRNA were cultured in 6-well plates for 24 h. The cells were then harvested after the cell suspension was centrifuged at 1,000 rpm for 5 min. After washing twice with PBS, the cells were resuspended in 400 µL of HEPES buffer and adjusted to a concentration of 1×10^6^ cells/mL. The assay was performed by a 2-colour analysis of FITC-labeled binding and PI uptake using the Annexin V-FITC apoptosis detection kit. After positioning the quadrants on the Annexin V/PI dot plots, live cells (Annexin V-/PI-), early/primary apoptotic cells (Annexin V+/PI-), late/secondary apoptotic cells (Annexin V+/PI+), and necrotic cells (Annexin V-/PI+) were distinguished. When calculating the total percentage of cells with fluorescence the Annexin V+/PI- and Annexin V+/PI+ cells were included. After the transfection with target shRNA or control shRNA and culturing in 6-well plates for 24 h., the cells were trypsinised and centrifuged for 5 min. The supernatant was removed and 195 mL of binding buffer and 5 mL of Annexin V-FITC were added. The cells were incubated for 10 min in the dark at room temperature and then centrifuged. After the supernatant removal, 190 mL of binding buffer and 10 mL of PI were added to the cell pellet, after which the cells were incubated for 5 min in the dark at room temperature. The fluorescence of 10,000 events per sample was analysed using flow cytometery (BD Immunocytometry Systems, San Jose, CA). After co-incubation with FITC-labeled annexin V (Becton–Dickinson, Franklin Lakes, NJ, USA) for 30 min in a dark, the cells was analyzed using a FACScan flow cytometer (Becton–Dickinson). The data were analyzed by CellQuest 3.0 software (Becton–Dickinson).

### Caspase-3 Activity Assay

The levels of caspase-3 activity in HSFBs were assayed using a commercial fluorometric assay kit (caspase colorimetric protease assay kit, MBL, Nagoya, Japan), according to the manufacturer’s instructions. Detached cells were collected, washed 3 times with cold PBS (0.01 M, pH 7.4), and suspended in 100 µL of cell lysis buffer. The cells were incubated at 4°C for 10 min, and the lysates were then centrifuged at 15,000 rpm for another 3 min. The supernatants were collected and incubated with 200 µmol/L Asp-Glu-Val-Asp-p-nitroanilide at 37°C for 24 h. The optical density for each specimen was determined at 405 nm using a plate reader (Bio-Rad Laboratories, Inc., Hercules, CA, USA) to measure the caspase-3 activity.

### Quantitative Real-time PCR Assays of Type I and III Precollagen

The type I and III precollagen mRNA expressions were analysed by quantitative real-time RT-PCR as in our previous study [13]. Total RNA was isolated from HSFBs using TRIzol reagent (Invitrogen Corporation). The levels of mRNA expression were quantified by real-time RT-PCR using a Takara SYBR1 EXScript RT-PCR kit (Takara Bio Inc.) and a 7300 real-time PCR system (Applied Biosystems, Foster City, CA). The annealing temperature was maintained at 56°C; the rest of the conditions included denaturation at 94°C for 30 s, followed by extension at 72°C for 40 s. These reactions were performed for 40 cycles. The primer sequences were used as in the following: type I precollagen, forward: 5′-CGGACGACCTGGTGAGAGA-3′′, reverse: 5′-CATTGTGTCCCCTAATGCCTT-3′′; type III precollagen, forward: 5′-CGAGGTAACAGAGGTGAAAGA-3′′, reverse: 5′-AACCCAGTATTC TCCACTCTT-3′; and β-actin, forward: 5′-CACCACCATGTACCCTGGCA-3′, reverse: 5′-GCTGTCACCTTCACCGTTCC-3′. A melting curve analysis was performed to demonstrate the specificity of each PCR product as a single peak. A control reaction containing all of the components except for the template was included in all experiments. The fold changes were finally calculated according to the 2^−ΔΔCT^ method for each transcript and were expressed relative to the values from the control group samples.

### Statistical Analysis

The data were expressed as mean±standard deviation. The differences between tissue groups were determined by the Mann-Whitney’s *U* test, and the correlation between SFRP2 and Slug was assessed by the Spearman’s rank correlation test; the *P*-values corresponded to 2-sided tests. The differences among cell groups were analyzed by one-way ANOVA with the SPSS 13.0 statistical software (SPSS Inc, Chicago, IL, USA). A *P*<0.05 was considered statistically significant.

## Results

### SFRP2 and Slug Expression were Increased in HS Compared with Normal Skin

**Figure 1:**
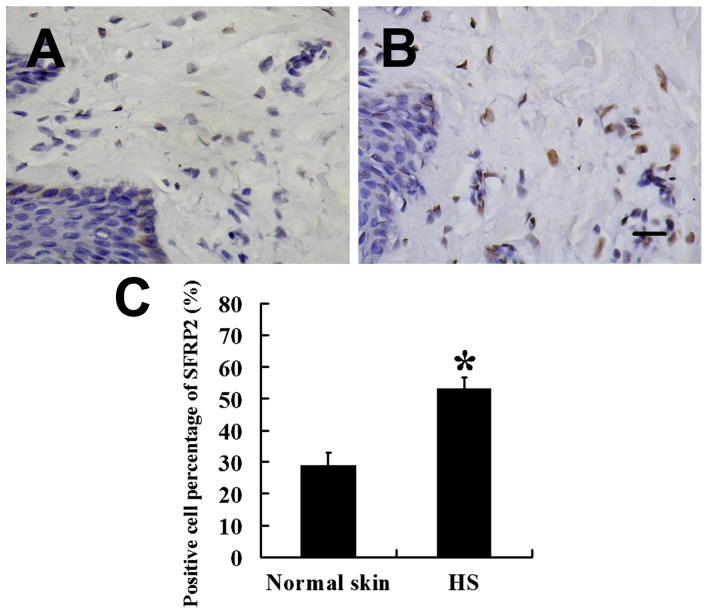
Expression of SFRP2 in HSFBs and normal skin fibroblasts. Expression of SFRP2 was significantly higher in HSFBs (B, 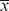 = 53.24, *s* = 3.33, *n* = 38) than that in normal skin fibroblasts (A, 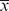 = 28.95, *s* = 3.89, *n* = 22). (C) Staining analysis of SFRP2 in HS and normal skin. Scale bar: 20 µm (A and B). * *P*<0.01.

**Figure 2:**
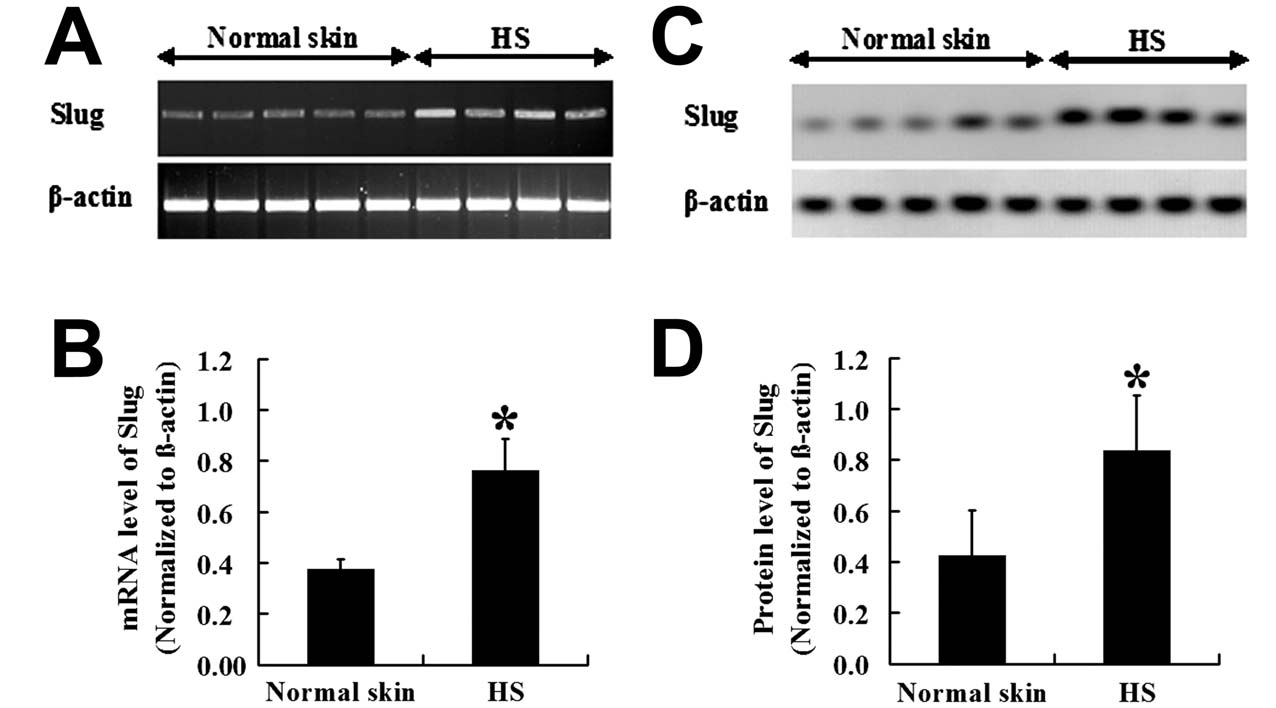
mRNA expression of Slug in HS and normal skin tissues. mRNA expression of Slug was increased in HS (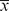 = 0.76, *s* = 0.13, *n* = 4) compared to normal skin (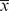 = 0.38, *s* = 0.04, *n* = 5) (A) and (B). Similar to the change of Slug mRNA level, western blot (C) and graphic analysis (D) showed that Slug was significantly increased in HS (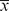 = 0.84, *s* = 0.22, *n* = 4) than that in normal skin (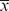 = 0.42, *s* = 0.18, *n* = 5). * *P*<0.01.

The immunohistochemical staining of SFRP2 is shown in Fig. 1. Cytoplasmic SFRP2 staining was observed in all the samples, and the SFRP2 expression was significantly stronger in HS (Fig. 1B) than in normal skins (*P*<0.01; Fig. 1A). To elucidate the correlation between Slug and fibroblasts apoptosis, The Slug mRNA expression in HS and that in normal skin was investigated by RT-PCR. The expression level of Slug mRNA was higher in HS than in normal skin (Figs. 2A and 2B). This was confirmed at the protein level; a high Slug expression was detected in 4 HSs, whereas a low Slug expression level was found in normal skin (Fig. 2C). The Slug expression level in HS and normal skin differed significantly (*P*<0.01; Fig. 2D).

**Figure 3:**
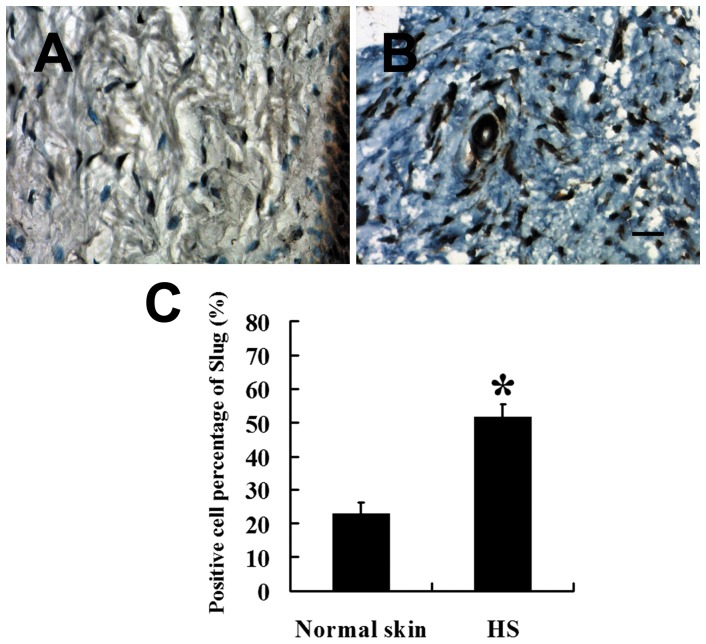
Expression of Slug in HSFBs and normal skin fibroblasts. Nuclear positive Slug was significantly higher in HSFBs (B, 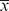 = 51.73, *s* = 3.74, *n* = 38) than that in normal skin fibroblasts (A, 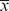 = 22.91, *s* = 3.33, *n* = 22). (C) Staining analysis of Slug in HS and normal skin. Scale bar: 20 µm (A and B). * *P*<0.01.

The immunohistochemical staining showed that the Slug expression level was also significantly higher in HS (Figs. 3A and C) than in normal skin (*P*<0.01; Figs. 3B and C). Furthermore, the presence of Slug was positively correlated with SFRP2 (*P*<0.01, OR = 0.837).

### shRNA Targeting SFRP2 Down-regulated Slug Expression

**Figure 4:**
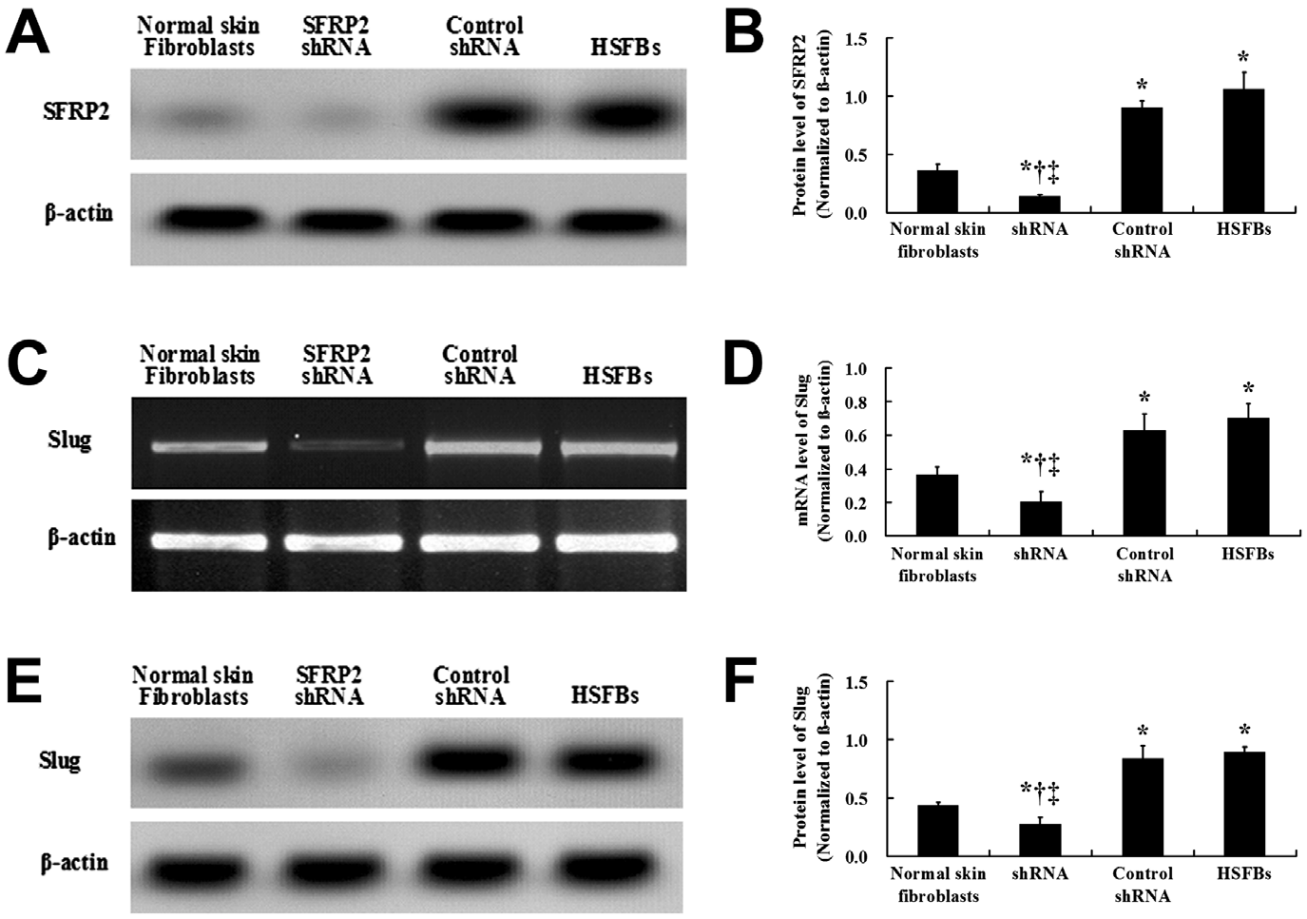
Effects of SFRP2 shRNA on the protein expression of SFRP2 and Slug in HSFBs. The SFRP2 protein level was significantly increased and decreased in the non- transfected HSFBs (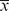 = 1.06, *s* = 0.15) and the SFRP2 shRNA group (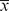 = 0.14, *s* = 0.02), respectively, compared with the normal skin fibroblasts (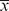 = 0.36, *s* = 0.05). And the protein expression of SFRP2 in HSFBs transfected with control shRNA (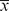 = 0.90, *s* = 0.06) was not significantly decreased than that in non-transfected HSFBs. After the treatments of the shRNAs, the SFRP2 protein level was significantly lower than that in the HSFBs and the HSFBs transfected with control shRNA (A and B). Similar to the effects on the expression of SFRP2, Slug expression was significantly higher in the non- transfected HSFBs and HSFBs transfected with control shRNA than that in normal skin fibroblasts both in mRNA (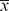 = 0.70, *s* = 0.08; 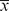 = 0.63, *s* = 0.10; 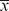 = 0.37, *s* = 0.05, respectively) and protein levels (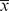 = 0.90, *s* = 0.04; 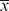 = 0.84, *s* = 0.11; 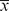 = 0.43, *s* = 0.04, respectively). Moreover, both the Slug mRNA and protein levels were significantly decreased in HSFBs transfected with SFRP2 shRNA (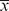 = 0.20, *s* = 0.06; 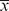 = 0.25, *s* = 0.05, respectively) compared with the non- transfected HSFBs and HSFBs transfected with control shRNA (B- F). *: *P*<0.01 versus Normal skin fibroblast; ^†^: *P*<0.01 versus Control shRNA; ^‡^: *P*<0.01 versus HSFBs.

Compared with that in the normal skin fibroblasts, the SFRP2 protein expression level was significantly increased in the HSFBs (Figs. 4A and B). Western blotting was used to assay the SFRP2 protein expression on the second day after the the transfection, suggesting that the shRNA targeting SFRP2 sufficiently suppressed SFRP2 expression. The control shRNA was also unable to significantly down-regulate the SFRP2 protein expression in the HSFbs.

After obtaining a positive correlation between the Slug and SFRP2 expressions in normal skin and HS tissues, we next tried to detect the mRNA and protein expressions of Slug after transfectionwith shRNA targeting SFRP2. The Slug mRNA and protein expression levels in the HSFBs transfected with SFRP2 shRNA were significantly decreased compared with those in the control shRNA treatment and untransfected HSFBs (Figs. 4C–F). The data demonstrated that transfection with SFRP2 shRNA led to significant down-regulation of Slug mRNAs and protein expression levels in HSFBs.

### shRNA Targeting Slug Promoted HSFB Apoptosis and Inhibited Bcl-2 mRNA and Protein Expressions

**Figure 5:**
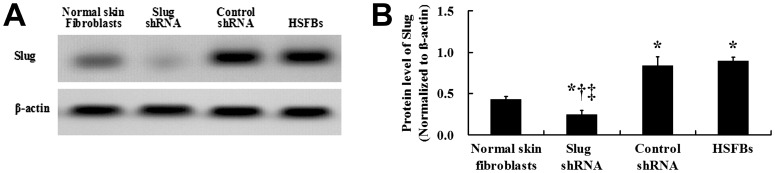
Effects of Slug shRNA on the protein expression of Slug in HSFBs. The Slug protein level was significantly decreased in the HSFBs of the Slug shRNA group (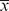 = 0.26, *s* = 0.05), compared with the normal skin fibroblasts (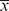 = 0.46, *s* = 0.04), HSFBs transfected with control shRNA (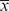 = 0.86, *s* = 0.10) and non-transfected HSFBs (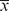 = 0.92, *s* = 0.04). And the protein expression of Slug in HSFBs transfected with control shRNA was not significantly decreased than that in non-transfected HSFBs. And the Slug protein level was significantly lower in normal skin fibroblast than that in non-transfected HSFBs and HSFBs transfected with control shRNA (A and B). *: *P*<0.01 versus Normal skin fibroblast; ^†^: *P*<0.01 versus Control shRNA; ^‡^: *P*<0.01 versus HSFBs.

The results of the western blotting suggested that targeted shRNA sufficiently suppressed the expression of Slug. The Slug protein expression level was significantly increased in the untransfected HSFB, compared with the normal skin fibroblasts (Figs. 5A and B).

**Figure 6:**
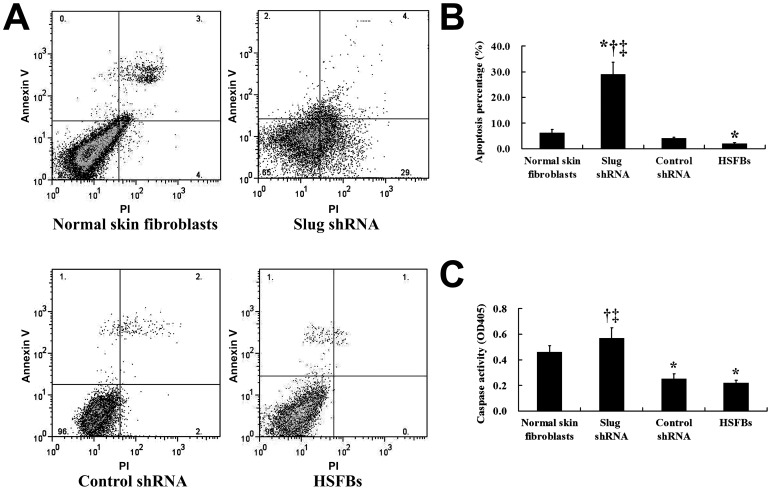
Effect of shRNA targeting Slug on the apoptosis and proliferation of HSFBs. FACS result suggested that the apoptosis percentage was significantly elevated after the treatment of shRNA targeting Slug (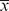 = 29.23, *s* = 4.72) compared with the non-transfected HSFBs (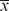 = 2.36, *s* = 0.34) and the control group (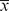 = 4.40, *s* = 0.50), respectively (A and B). In addition, shRNA targeting Slug markedly enhanced the activity of apoptosis related signal molecule caspase-3 (C), further supporting that Slug suppressed the apoptosis of HSFB, which ultimately resulted in the HS formation.

The FACS result demonstrated that the shRNA suppression of Slug significantly promoted HSFB apoptosis compared with the control shRNA transfection (Figs. 6A and B). Moreover, we also measured the apoptosis-related molecule caspase-3 by enzyme fluorometric assay. Caspase-3 activity was increased in HSFBs, after transfection with Slug shRNA, which may demonstrate that the suppression of Slug in HSFBs cells could induce apoptosis (Fig. 6C).

**Figure 7:**
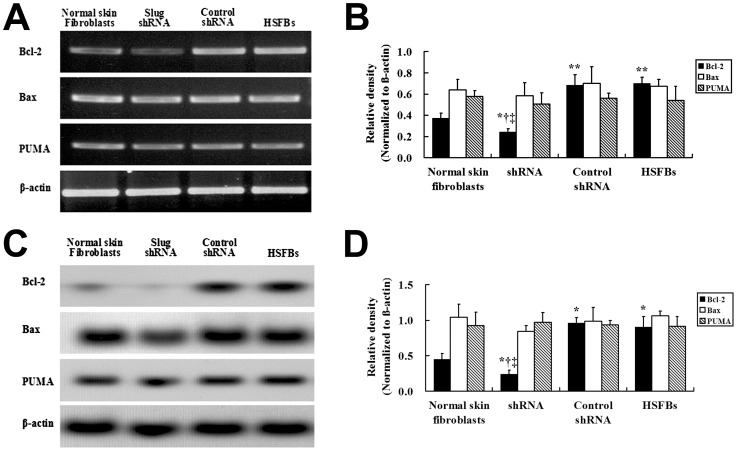
Effects of Slug shRNA on the expression of apoptosis-relative genes in HSFBs. The mRNA expression of Bcl-2 is decreased in HSFBs transfected with Slug shRNA (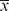 = 0.23, *s* = 0.03) than those in other groups. HSFBs transfected with control shRNA (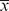 = 0.68, *s* = 0.10) and non-transfected HSFBs (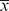 = 0.70, *s* = 0.06) expressed the most increased level of Bcl-2 than that in normal skin fibroblasts (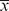 = 0.38, *s* = 0.05) and HSFBs transfected with Slug shRNA (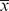 = 0.23, *s* = 0.03) (A and B). Similar with mRNA expression level, the protein expression of Bcl-2 is most decreased in Slug shRNA group (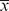 = 0.24, *s* = 0.06) and most increased in control shRNA (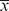 = 0.96, *s* = 0.07) and non-transfected group (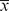 = 0.90, *s* = 0.15) (C and D). Expression of Bax and PUMA at mRNA and protein level was detected in all groups. The mRNA level of Bax and PUMA was similar among the four groups (A and B). Similarly, western blot (C) and graphic analysis (D) showed that Bax and PUMA were similar in all groups.*: *P*<0.01 versus Normal skin fibroblast; ^†^: *P*<0.01 versus Control shRNA; ^‡^: *P*<0.01 versus HSFBs.

Given the known role of Slug in inhibitting apoptotisis by regulating the Bcl-2, Bax expression and the transactivation of Puma in some tumours [14]; [15], the Bcl-2, Bax, and PUMA mRNA were assayed by RT-PCR (Fig. 7A) and the proteins were identified by western blot (Fig. 7C). The Bcl-2 mRNA expression level was lower in the HSFBs after the transfection with Slug-targeted shRNA than in the other experimental groups (Figs. 7A and B). This was confirmed at the protein level (Figs. 7C and D). However, there was no significant difference in Bax and PUMA mRNA or protein levels between the 4 groups.

**Figure 8:**
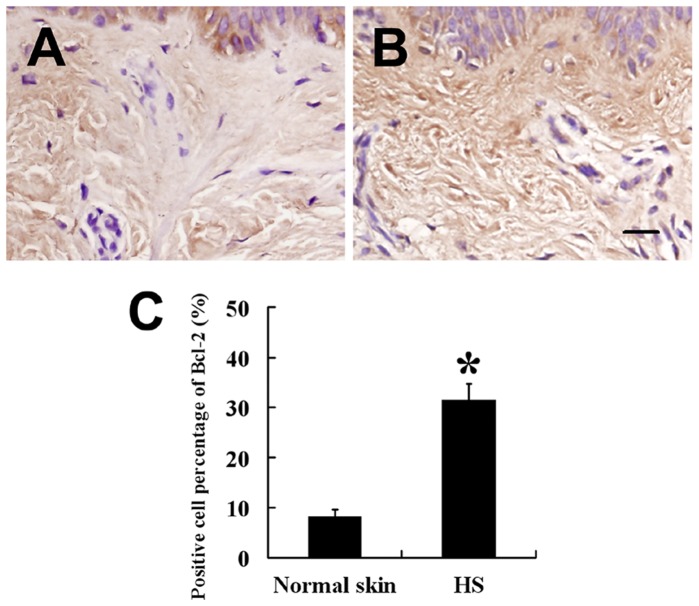
Expression of Bcl-2 in HSFBs and normal skin fibroblasts. Expression of Bcl-2 was significantly higher in HSFBs (B, 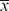 = 31.54, *s* = 3.19, *n* = 38) than that in normal skin fibroblasts (A, 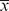 = 8.12, *s* = 1.58, *n* = 22). (C) Staining analysis of Bcl-2 in HS and normal skin. Scale bar: 20 µm (A and B). * *P*<0.01.

To confirm Bcl-2 expression pattern in hypertrophic scars, we detected the Bcl-2 staining in the tissues. Immunohistochemically, expression of Bcl-2 was also significantly higher in hypertrophic scars (Fig. 8B) than in normal skins (Fig. 8A).

### Slug-targeted shRNA had no Effect on the mRNA Levels of Type I and III Procollagen

**Figure 9:**
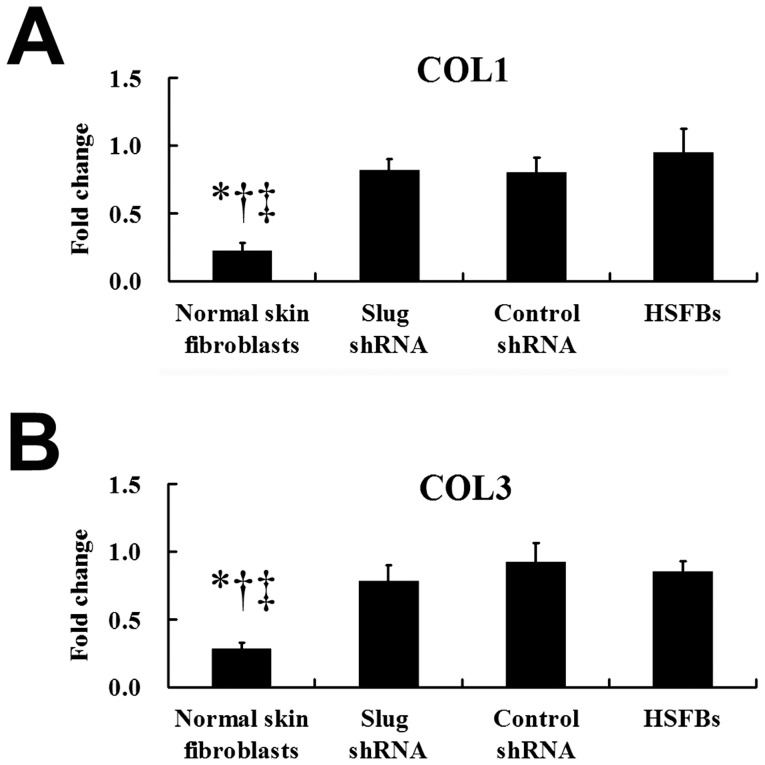
Quantitative RT-PCR analysis of COL1 and COL3 mRNA expressions . The mRNA levels of COL1 (A) and COL3 (B) in normal skin fibroblasts (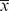 = 0.24, *s* = 0.06; 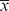 = 0.28, *s* = 0.05, respectively) were significantly lower, compared with other three groups. However, there is no significant difference in mRNA levels of COL1 (A) and COL3 (B) in HSFBs transfected with Slug shRNA (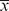 = 0.82, *s* = 0.08; 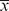 = 0.78, *s* = 0.12, respectively), compared with non-transfected HSFBs (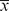 = 0.95, *s* = 0.17; 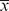 = 0.85, *s* = 0.08, respectively) and HSFBs transfected with control shRNA (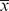 = 0.80, *s* = 0.11; 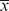 = 0.92, *s* = 0.14, respectively), suggesting Slug shRNA had no effect on the collagen proteins synthesis in HS formation. *: *P*<0.01 versus Slug shRNA; ^†^: *P*<0.01 versus Control shRNA; ^‡^: *P*<0.01 versus HSFBs.

In normal skin fibroblasts, the mRNA level of COL1 or COL3 was significantly lower, compared with that in HSFBs (Fig. 9). Given that HS is characterised by excessive collagen synthesis and our previous study demonstrated that HSFBs transfected with SFRP2 shRNA led to significant down-regulation of type I and III procollagen mRNAs, we assessed COL1 and COL3 mRNA levels after the treatment with shRNA targeting Slug. However, there was no significant difference in COL1 or COL3 mRNA levels between the HSFBs transfected with Slug shRNA and the untransfected HSFBs (Fig. 9), suggesting that Slug-targeted shRNA had no effect on the mRNA levels of type I and III procollagen.

## Discussion

Hypertrophic scars result from alterations in the normal processes of cutaneous wound healing. In normal wound healing, the scar develops from the granulation tissue, which is rich in cells, and as the wound becomes epithelialised, the number of cells undergoing apoptosis increases, suggesting that apoptosis is an important mechanism responsible for the evolution of granulation tissue into a normal scar [20]. HS can be considered a granulation tissue with poor cellular apoptosis. Recently, studies on fibroblast apoptosis have been focused on the formation of HS [5]; [7]. Moreover, apoptosis or apoptosis-associated proteins were demonstrated to be increased or to be maintained at the same level in pathological scars compared with normal skins [21].

SFRP2 is a member of the SFRP family of secreted apoptosis-related proteins. Our previous studies investigated the dramatic up-regulation of the SFRP2 mRNA level in HS tissue [12], and the sharp increase in primary fibroblasts apoptosis upon suppression of SFRP2 [13]. Accordingly, in this study, we confirmed by immunohistochemistry that SFRP2 protein level were notaby increased in HS compared with normal skin, which supports the importance of SFRP2 in HS formation. However, in contrast to this proposal, the research of Russell et al [22] suggested that in keloids, a type of dermal fibrosis, the expression of SFRP2 was significantly decreased. Moreover, another study [23] indicated that SFRP2 is profibrotic but also proapoptotic in the injured region of the heart. This contradiction implies that despite its important roles in the regulation of fibroblast apoptosis, SFRP2 modulation of cellular apoptosis is tissue-specific and related to the complex extrinsic microenvironment.

The underlying mechanisms by which SFRP2 affects HS formation are currently unclear. Research has demonstrated that the inhibition of fibroblast apoptosis and proliferation promotion are key processes in the formation of HS [1]; [5]. The proliferation level of HSFBs was higher than that of normal skin fibroblasts, and some studies have shown that SFRP2 may also directly modulate cell proliferation in other diseases [24]. Our previous study [13], however, failed to observe that SFRP2 shRNA had a significant suppression effects on the proliferation of HSFBs, suggesting that SFRP2 was not associated with the proliferation of HSFBs. Moreover, consistent with some studies [1]; [5]; [6]; [7] on fibroblast apoptosis during HS formation, our previous results showed that SFRP2 suppression also up-regulated the apoptosis-related molecule caspase-3, implying that SPRP2 regulates fibroblast apoptosis at least partially through caspase-3-dependent pathway.

The transcription factor, Slug, has been indicated to inhibit cellular apoptosis in tumours and other cells [14]; [15]; [25]. A study on Slug-null mice revealed that functional classification of altered gene expression was consistent with a role for Slug in keratinocyte development and differentiation, and apoptosis [17]. It is interesting that in our study, the expression of Slug was notably increased in the HS tissues compared with the normal skin tissues, which may imply the involvement of Slug in HS formation. Moreover, a previous study demonstrated that SFRP2 plays important roles in the regulation of Slug [18]. Consistent with this, a positive correlation between Slug and SFRP2 expression was found in our study, and we assumed that SFRP2 shRNA would affect the Slug expression in HSFBs. In fact, shRNA targeting SFRP2 decreased Slug expression.

Previous studies revealed that Slug expression promotes proliferation and inhibits cell apoptosis in vitro and in vivo [26]. In this study, we used shRNAs targeting Slug to characterise Slug function in HSFBs, including role in apoptosis and proliferation. An intriguing, finding is that the HSFB apoptosis rate was significantly elevated after the down-regulation of Slug expression by RNA interference; in addition, caspase-3 activity in the HSFBs was sharply enhanced after the knockdown of Slug. This, together with results from tissues, indicates that the high level of Slug contributed to the anti-apoptotic mechanism of the fibroblasts in HS, which may affect HS formation. There was no significant suppression effect on the proliferation of HSFBs after the treatment with Slug shRNA (data not shown), although directly modulation of the cell proliferation effects of Slug in other cells was demonstrated in other cell types in previous studies [27]; [28]. Therefore, the predominant role of Slug in HS formation is primarily related to the regulation of cellular apoptosis.

Normally, apoptosis-related genes such as Bcl-2 could also mediate apoptosis in fibroblasts, hypertrophic scars, and active keloid tissues. Intense Bcl-2 staining is detected in basal keratinocytes and in scattered fibroblast-like cells [8]. Coinciding with previous study, here we found a total increase in Bcl-2 expression level in the untransfected HSFBs compared with normal skin fibroblasts. Moreover, Bcl-2 expression was increased in HS tissues compared with normal skin tissues. It is interesting that Slug was involved in cell survival through the direct or indirect transcriptional regulation of anti-apoptotic genes [29]. In our studies, the anti-apoptotic gene, Bcl-2, was detected in the fibroblasts, and a sharp decrease in Bcl-2 expression in the Slug shRNA-transfected HSFBs (compared with the untransfected HSFBs) demonstrates that Slug plays its anti-apoptotic role in HS formation through Bcl-2. Although other apoptosis-relatived genes such as Bax and PUMA were reported to be regulated by Slug [30]; [31], there was no difference in Bax and PUMA expressions in this study among the 4 groups. Although the mechanisms by which Slug regulates cellular apoptosis in HS formation are currently unclear, it appears that Slug performs its antiapoptotic function primarily through regulation of Bcl-2 levels.

HS is characterised by an excessive production and deposition of collagen. Recent studies have indicated that collagen content changes in HS [32]. In our previous study, we detected the mRNA levels of COL1 and COL3 in normal skin fibroblasts, the mRNA levels of COL1 or COL3 were significantly lower, compared with that in HSFBs. Moreover, we found that SFRP2 suppression down-regulated type I and III procollagen expressions and the inhibition of type III procollagen expression was more significant, suggesting that SFRP2 was attributed to collagen protein synthesis [13]. Consistent with our observation, Kobayashi et al. [23] demonstrated that SFRP2 enhanced collagen deposition and fibrosis in the infarcted heart. Conversely, in another study, SFRP2 injection resulted in an overall reduction of collagens in the myocardium in vivo, suggesting a role for SFRP2 in inhibiting fibrosis [33]. In this study, we found that SPRP2 would affect the expression of Slug in HSFBs; to investigate the possibility of a role for Slug in collagen synthesis in HSFBs, we investigated the expressions of type I and III procollagen in HSFBs transfected with Slug-targeted shRNA. However, we failed to find that either type I or III procollagen expression was affected by Slug suppression. It appears that SFRP2 regulates the deposition of collagen in HS through other factor(s).

Although HS formation is a highly complex and organized process that consists of multiple interrelated steps, SFRP2 appears to interact with Slug to affect the apoptosis of hypertrophic scar fibroblasts. Though overexpression of SFRP2 or Slug in normal skin fibroblasts had no significant effect on the cellular apoptosis or deposition of collagen (Data was not shown), this may suggest that SFRP2 and Slug are the key molecules but not the only ones in HS formation. Given that SFRP2 and Slug down-regulation may represent a novel approach to prevent HS formation, understanding the molecular mechanism behind this regulation by SFRP2 will contribute to the dissection of factors underlying HS formation.
